# Exploring new therapeutic drugs for osteoarthritis and osteoporosis: Glucagon-like peptide-1 receptor agonists: A review

**DOI:** 10.1097/MD.0000000000043239

**Published:** 2025-07-18

**Authors:** Meiqi Zheng, Jingjing Zhao, Yuxuan Wang, Zifan Cui, Zihong Qiao, Hongzhuo Wu, Chenxia Shi, Xiaofeng Wang

**Affiliations:** a Department of Pharmacology, Hebei Medical University, Shijiazhuang, Hebei, China; b Department of Orthopaedic Surgery, Hebei Medical University Third Hospital, Shijiazhuang, Hebei, China.

**Keywords:** bone, cartilage, glucagon-like peptide-1 receptor agonist, osteoarthritis, osteoclasis, osteogenesis, osteoporosis

## Abstract

As the 2 most common diseases of bone and joint, osteoarthritis (OA) and osteoporosis (OP) have a high incidence. The two diseases are often accompanied in clinical practice, affecting the health and quality of life of patients, but the relationship is complex. So far, there are no very effective drugs for the treatment of both diseases, so it is very meaningful to search for drugs that are suitable for OA and OP. Glucagon-like peptide-1 (GLP-1), as a type of enterotropic insulin, has hypoglycemic and weight loss effects. Recently, the effects of GLP-1 receptor agonists (GLP-1RA) on OA and OP have attracted more and more attention. This review aims to summarize the mechanism of GLP-1RA affecting bone metabolism in OA and OP, such as anti-inflammatory, affecting chondrocyte matrix metabolism, analgesia, promoting bone formation, inhibiting bone resorption, and reducing fracture incidence, to provide new methods for drug treatment of OA and OP.

## 1. Introduction

Osteoarthritis (OA) and osteoporosis (OP) are highly prevalent as 2 of the most common bone and joint diseases. OA is a chronic degenerative joint disease characterized by articular cartilage degeneration, subchondral bone remodeling, synovitis, and joint muscle atrophy. Moreover, it is estimated that 596 million people worldwide will suffer from OA by 2020, which is equal to 7.6% of the global population^.[1]^ OP is a systemic bone disease characterized by microarchitectural bone disruption, low bone mineral density (BMD), increased bone fragility, and fracture susceptibility. In 2021, a meta-analysis of epidemiological trends in OP over the past 20 years showed that the global prevalence of OP is approximately 18.3% and 23.1% in women and 11.7% in men.^[2]^ OA and OP often coexist, and the relationship between the 2 diseases is complex. OP reduces the incidence of knee and hip OA,^[3]^ whereas Zheng et al.^[4]^ reported that OP aggravates OA symptoms. The 2 diseases, OA and OP, occur mostly in middle-aged and elderly people and can cause disability and significantly reduce the quality of life. To date, there are no effective drugs that can treat both diseases, and it is important to search for drugs that are suitable for OA and OP.

Glucagon-like peptide-1 (GLP-1) is an enteric insulin that stimulates insulin secretion, inhibits glucagon secretion, delays stomach emptying, suppresses appetite, reduces food intake, and lowers blood glucose^[5]^ and weight.^[6]^ However, natural GLP-1 easily degrades and eliminates its activity; therefore, researchers have developed a GLP-1 receptor agonist (GLP-1RA) that mimics the action of GLP-1 by binding to its receptor. GLP-1RA is not easily degraded and can prolong the duration of action. Currently, GLP-1RAs include exenatide, liraglutide, semaglutide, dulaglutide, albiglutide, beinaglutide, and lixisenatide. Studies have reported that GLP-1RA can reduce the risk of OA and OP in patients and has a positive effect on the treatment of OA and OP.^[7,8]^ This study aims to review the role of GLP-1RA in OA and OP, and provides a new method for the pharmacological treatment of the two diseases.

## 2. Application of GLP-1RA in OA

GLP-1R is expressed in cartilage chondrocytes in humans and animals and decreased in OA cartilage.^[9–11]^ Compared with the control group, the blood GLP-1 content of patients with OA was reduced, and the level of GLP-1 in the synovial fluid of the knee joint was positively correlated with the plasma GLP-1 level. Additionally, intra-articular injection of liraglutide mitigated cartilage degradation in OA mice.^[9]^ Clinical studies have demonstrated that patients with type 2 diabetes who received GLP-1RAs therapy attenuated the risk of knee OA and total knee replacement compared with non-GLP-1RA-treated patients.^[7]^ GLP-1R may serve as a potential therapeutic target for OA. In this review, the effects of GLP-1RAs on OA (shown in Table 1) regarding their anti-inflammation effects, influence on matrix metabolism of chondrocytes, and analgesia.

**Table 1:** Effects of GLP-1RA on OA.

Drug	Model	Results	Conclusion	Reference
The impact on inflammatory factors				
Liraglutide	IL-1β treated-chondrocytes	Cleaved-caspase3, Bax, GRP78, CHOP, PDI, caspase12↓; BCL-2↑;	Inhibit cell apoptosis; inhibit ER stress	[10]
	TG-treated chondrocytes	TNF-α, IL-6↓	Anti-inflammatory	
	Cut off ACL transection combined with medial menisci resection on the rat's right knee joint	CHOP, cleaved-caspase3, OARSI score↓	Inhibit cell apoptosis; anti-inflammatory	
Liraglutide	Injected MIA OA mice model	Improve synovitis severity score	Anti-inflammatory	[11]
	IL-1β induced primary chondrocytes	IL-6, PGE2, COX-2, iNos, Cox-2, TNF-α↓	Anti-inflammatory	
	LPS-induced mouse macrophage RAW264.7	NO, PGE2, IL-6, Cox-2, TNF-α, MCP-1, Cd38↓; Erg-2↑	Anti-inflammatory	
Liraglutide	Intra-articular injection of MIA in rats	IL-6, TNF-α, IL-1β↓	Anti-inflammatory	[12]
Liraglutide	LPS or IL-1β induced equine primary chondrocytes	0.5 μM and 8 μM Liraglutide reduced CCL2 by 70% and 62%, respectively	Anti-inflammatory	[13]
	Acute synovitis in equine induced by LPS	GAG, MMP-activity, CPII, and C2C None	Does not improve inflammatory	
Liraglutide	LPS-induced mouse macrophage RAW264.7	PGE2, IL-6, TNF-α↓; IL-10, MRC-1, ARG-1↑	Anti-inflammatory	[14]
Liraglutide	Age-induced primary chondrocytes of rats	IL-1β, IL-6, IL-12, TNF-α↓	Anti-inflammatory	[15]
Liraglutide	TNF-α-stimulated human primary chondrocytes	IL-6, MCP-1, ROS, NOX-4, TNF-α↓	Anti-inflammatory; attenuate TNF-α-induced oxidative stress	[16]
Dulaglutide	AGEs induced human SW1353 cell line	IL-6, IL-8, MCP-1, PGE2, COX-2, ROS↓	Anti-inflammatory; antioxidant stress	[17]
Lixisenatide	AGEs induced primary human chondrocytes	4-HNE, NOX-4, TNF-α, IL-6 ↓; mitochondrial membrane potential, ATP↑	Protect mitochondrial function; reduce oxidative stress; anti-inflammatory	[18]
Exenatide	Macrophages differentiated from CD14 + monocytes	STAT3 activation; IL-10↑	Promote macrophage polarization toward the M2 phenotype	[19]
Exenatide	AGEs induced human primary chondrocytes	TNF-α, IL-1β↓	Anti inflammatory	[20]
Effect on matrix metabolism of chondrocytes				
Liraglutide	TG-treated chondrocytes	Collagen↑; MMP-3↓	Attenuate the ECM catabolic activity	[10]
Liraglutide	IL-1 β-induced primary chondrocytes	ADAMTS-4, ADAMTS5, MMP-3, MMP-13↓	Resistant to ECM degradation	[11]
Liraglutide	Age-induced primary chondrocytes of rats	MMP-3, MMP-13, ADAMTS-4, ADAMTS-5↓; CollⅡ, aggrecan↑	Inhibit catabolism; promote anabolism	[15]
Liraglutide	TNF-αinduced human primary chondrocytes	MMP-3, MMP-13, ADAMTS-4, ADAMTS-5↓; Aggrecan and collagen II degradation decreased from 75% to 25% and from 50% to 20%, respectively	Inhibit ECM degradation	[16]
Dulaglutide	AGEs induced human SW1353 chondrocytes	MMP-3, MMP-13↓; the degradation of CollⅡand aggrecan↓	Inhibit ECM degradation	[17]
	AGEs treatment of chondrosarcoma chondrocytes in human SW1353 cell line	p65 protein, luciferase activity of NF-κB↓	Protect cartilage from damage	
Lixisenatide	AGEs induced primary human primary chondrocytes	MMP-3, MMP-13, ADAMTS-4, ADAMTS-5↓ The degradation of ColⅡ and aggrecan↓	Inhibit ECM degradation	[18]
Exenatide	AGEs induced human primary chondrocytes	ROS, GSH, p38↑; MMP-3, MMP-13, ADAMTS-4, ADAMTS-5, the degradation of CollⅡand aggrecan↓	Reduce oxidative stress; Inhibit ECM degradation	[20]
The impact on pain				
Liraglutide	Injected MIA OA mice model	Improve the synovitis severity score	Analgesic	[11]
Liraglutide	LPS-induced equine acute synovitis	No improvement in gait asymmetry or pain score	No reduction in pain	[19]
Liraglutide	Overweight/obese patients with knee OA following an 8-week low-calorie diet intervention	Lose weight; knee injury and Osteoarthritis Outcome Score (KOOS) no change	No reduction in knee pain	[21]
Semaglutide, Dulaglutide, Liraglutide, etc.	KOA patients with type 2 diabetes	Surgery incidence: 1.7% (GLP-1RA) vs. 5.9% (non-GLP-1RA); Improve WOMAC Pain score	Relieve pain	[22]
		Annual consumption before treatment vs after treatment (oral nonsteroidal anti-inflammatory drugs and acetaminophen): 16.8 ± 14.7 vs 14.0 ± 12.2	Analgesic	
		Annual consumption of nonsteroidal anti-inflammatory drugs before and after treatment: 24.5 ± 24.3 vs 16.6 ± 19.6	Analgesic	
		Annual consumption of opioid drugs before and after treatment: 97.7 ± 195.1 vs 93.5 ± 182.3	Analgesic	
Semaglutide	Obese patients with moderate KOA and moderate to severe pain	WOMAC pain and function scores (41.7 vs 27.5, 41.5 vs 26.7)↓; The overall usage of opioid and codeine↓	Relieve pain	[23]
Dulaglutide	Elderly patients with type 2 diabetes combine with OA	VAS scores↓; NSAID consumption↓	Relieve pain	[24]
Other				
Exenatide, Liraglutide, Semaglutide, Dulaglutide, Albiglutide, Lixisenatide	Type 2 diabetes mellitus patients	KOA/TKR incidence rate↓		[7]
Semaglutide, Dulaglutide Liraglutide, etc.	Obesity status (BMI ≥ 30 vs < 30) Diabetic status (type 2 diabetes vs non-type 2 diabetes)	Incidence of hip and knee OA↑	No reduction in incidence	[25]

ACL = anterior cruciate ligament, ADAMTS4 = a disintegrin and metalloproteinase with thrombospondin motifs 4, ADAMTS5 = a disintegrin and metalloproteinase with thrombospondin motifs 5, AGE = advanced glycation end products, ARG-1 = arginine-1, ATP = Adenosine Triphosphate, Bax = BCL2- a ssociated x, BCL = B-cell lymphoma, caspase = cysteine-dependent aspartate-specific protease-12, CCL-2 = C-C motif chemokine ligand 2, Cd38 = Cluster of Differentiation 38, C/EBP = C/enhancer-binding protein, CHOP = C/enhancer-binding protein homologous protein, Coll = Collagen Type II, COX-2 = cyclooxygenase-2, CPII = C-Propeptide of Type II, ECM = extracellular matrix, ER = endoplasmic reticulum, Erg-2 = early response gene 2, GAG = glycosaminoglycan, GRP78 = glucose-regulated protein 78, HNE = hydroxynonenal, IL = interleukin, iNos = inducible nitric oxide sythase, LPS = lipopolysaccharide, MCP = Monocyte chemoattractant protein-1, MIA = monoiodoacetate, MMP = matrix metalloproteinase, MRC-1 = mannose receptor type C-1, NF-κB = nuclear factor kappa-B, NOX-4 = nicotinamide adenine dinucleotide phosphate oxidase-4, OARSI = Osteoarthritis Research Society International, PDI = protein disulfide isomerase, PGE2 = prostaglandin E2, RAW264.7 = mouse mononuclear macrophages cells, ROS = reactive oxygen species, STAT3 = signal transducer and activator of transcription 3, TG = triglyceride, TNF-α = tumor necrosis factor-alpha, WOMAC = Western Ontario and McMaster Universities Osteoarthritis Index.

### 2.1. Anti-inflammatory effects of GLP-1RA

OA is a chronic degenerative disease characterized by low-grade inflammation. Accumulating evidence indicates that GLP-1RAs activate GLP-1R in the articular cartilage and reduce the inflammatory response by influencing multiple intracellular signaling pathways. Que et al.^[12]^ reported that in a rat knee joint OA model induced by the intra-articular injection of monoiodoacetate (MIA), the expression of GLP-1R was significantly decreased, the protein kinase A (PKA)/cyclic adenosine monophosphate response element-binding protein (CREB) signaling pathway was downregulated, and the inflammation-related proteins were upregulated in cartilage tissue. Subcutaneous injection of GLP-1RA, liraglutide, upregulated the level of GLP-1R, increased the expressions of PKA, p-PKA, CREB, and p-CREB, and inhibited the expressions of tumor necrosis factor-α (TNF-α), interleukin-1β (IL-1β), and IL-6. Another study confirmed that liraglutide exerts anti-inflammatory effects on primary murine chondrocytes in a dose-dependent manner. In vitro, liraglutide administration significantly reduced IL-6, prostaglandin E2 (PGE2), and nitric oxide secretion in chondrocytes induced by IL-1β stimulation. However, treatment with the GLP-1R competitive antagonist exendin 9–39 (100 nM) completely reversed the anti-inflammatory effects of 50 nM liraglutide.^[11]^ In addition to its anti-inflammatory effects, GLP-1RA exhibits antiapoptotic effects by reducing endoplasmic reticulum stress. In IL-1β-induced chondrocytes, liraglutide activated the phosphatidylinositol 3-kinase/protein kinase B signaling pathway through GLP-1R, inhibited the expressions of endoplasmic reticulum stress-related proteins, such as glucose-regulated protein 78, protein disulfide isomerase, cystein-dependent aspartate specific protease 12 (caspase-12), and transcription factor C/enhancer-binding protein homologous protein, significantly reduced the levels of pro-apoptotic indicators B-cell lymphoma-2 associated x and cleaved caspase 3, increased the level of antiapoptotic protein B-cell lymphoma-2, thereby ultimately inhibited chondrocyte apoptosis.^[10]^

GLP-1 receptor is expressed in synovial membranes in humans and animals.^[11,13]^ Under physiological conditions, the main function of macrophage-like synovial cells is to phagocytose bacteria and debris produced by apoptotic cells, and maintain the dynamic balance between pro-inflammatory and anti-inflammatory factors in the synovial fluid. GLP-1RAs can transform polarized macrophages from a pro-inflammatory M1 phenotype to an anti-inflammatory M2 phenotype, thus alleviating inflammation. In vitro treatment of liraglutide significantly reduced the secretion of NO, PGE2, and IL-6 in lipopolysaccharide (LPS)-induced RAW 264.7 macrophages in a concentration-dependent manner. It was also confirmed that liraglutide improved the severity of synovitis in short-term MIA mouse model of OA.^[11]^ Exenatide induces polarization of monocyte-derived macrophages towards the M2 phenotype by activating transcriptional activator 3 (Signal Transducer and Activator of Transcription 3 [STAT3]).^[19]^ Further studies have reported that recombinant human GLP-1 (recombinant GLP-1) interfered with the cyclic adenosine monophosphate/PKA (cAMP/PKA) signaling pathway in RAW264.7 macrophages, inhibited the activation of Jun N-terminal kinase (JNK), reduced the content of p-JNK, and increased the level of p-STAT3, therefore regulated the polarization of macrophage. The results showed that the expression of M2-specific genes increased, such as IL-10, mannose receptor type C-1, macrophage galactose type 1, and arginine-1 (ARG-1) as well as the expression levels of M1-related genes, which were downregulated.^[14]^

Unlike the above-mentioned studies, Scheike et al^[13]^ found that although liraglutide in vitro reduced the production of inflammatory biomarker C-C motif chemokine ligand 2 in LPS-stimulated equine synovial cells, lilarlutide injected into the intercarpal joints failed to significantly alleviate the increase of synovial fluid biomarkers related to inflammation in the equine model of acute synovitis. We speculate that this difference can be attributed to the following reasons. First, the acute and high-intensity inflammatory response triggered by LPS fundamentally differs from the chronic and low-intensity inflammatory characteristics of OA. Second, compared with the extended dosing regimen used in the OA study, a single intra-articular injection may lead to insufficient drug exposure. Third, there may be differences in the regulation of drug metabolism and inflammatory pathways among species.^[13]^

### 2.2. Effect of GLP-1RA on chondrocyte matrix metabolism

The articular cartilage is the first and most important site of invasion when OA lesions occur. Articular cartilage consists of chondrocytes (the only cells that synthesize the cartilage matrix) and extracellular matrix (ECM). Type II collagen (Col II) and aggrecan are the main components of articular cartilage and are degraded by matrix metalloproteinases (MMPs, mainly MMP-3, MMP-8, and MMP-13, etc), and disintegrins and MMPs with thrombospondin motifs (a disintegrin and metalloproteinase with thrombospondin motifs [ADAMTSs], mainly ADAMTS-4 and ADAMTS-5). When OA occurs, various pathological factors act directly or indirectly on chondrocytes, causing an imbalance in the anabolic and catabolic processes of the ECM, ultimately leading to cartilage destruction.

Studies have implicated that the pro-inflammatory cytokines, particularly IL-6, IL-1β, and TNF-α, can promote the release of MMPs and ADAMTS, reduce the synthesis of Col II and aggrecan, destroy the ECM of chondrocytes, and eventually lead to OA by activating the STAT3 signaling pathway^[26]^ or the nuclear factor kappa-B (NF-κB) pathway.^[27]^

In vivo and in vitro experiments have confirmed that GLP-1RAs protect the cartilage by inhibiting catabolism and enhancing chondrocyte anabolism liraglutide reduced the mRNA and protein expressions of MMP-1, MMP-3, MMP-13, ADAMTS-4, and ADAMTS-5, while increasing the expressions of Col II and aggrecan whether in mouse chondrocytes stimulated by IL-1β,^[11]^ rat chondrocytes treated with triglycerides,^[10]^ rat chondrocytes induced by advanced glycation end products (AGEs),^[15]^ or in TNF-α-stimulated human chondrocytes.^[16]^ Overall, the experiment showed that after liraglutide was administered at 50 µg/kg/day subcutaneously and continued for 3 and 6 weeks, histopathological staining and Osteoarthritis Research Society International score indicated that liraglutide treatment significantly reduced cartilage destruction in the rat OA model.^[10]^

Oxidative stress can cause reduction-oxidation imbalance and increase ECM degradation due to the accumulation of reactive oxygen species (ROS) in chondrocytes. Activation of nicotinamide adenine dinucleotide phosphate oxidase-4 (NOX-4) is one of the main sources of ROS production.^[28]^ Numerous studies have reported the antioxidant effects of GLP-1RAs. For example, liraglutide downregulated ROS and NOX-4 production.^[16]^ In AGEs-stimulated human SW1353 chondrocytes, dulaglutide inhibited ROS production, reduced ECM decomposition, and this effect was mediated through the NF-κB pathway.^[17]^ Lixisenatide reduced the expression of lipid peroxidation hydroxynonenal (4-HNE) and NOX-4 in AGE-induced human primary chondrocytes.^[18]^ Exenatide activated the p38 cellular signaling pathway, which led to a reduction of ROS, enhanced the reduction of glutathione, and inhibited the activity of NF-κB luciferase.^[20]^

### 2.3. Analgesic effect of GLP-1RA

Joint pain is the main symptom of OA and seriously affects the quality of life of patients. The pathogenesis of OA pain is complex and originates from various joint tissues (such as synovium, subchondral bone, meniscus, ligaments, joint capsules, and periosteum, etc.). Pain is the response of these tissues to various types of harmful stimuli (such as mechanical stimulation, cold and hot stimulation, and chemical stimulation and is mainly caused by inflammatory mediators, etc.).

Currently, whether GLP-1RA has an analgesic effect on OA remains controversial.^[29]^ Meurot et al^[11]^confirmed that intra-articular injection of liraglutide alleviated pain-related behaviors and exerted an analgesic effect in an MIA-induced OA mouse model. The results of the von Frey test showed that compared with the vehicle-treated group, there was a significant dose-dependent increase in paw withdrawal threshold in the liraglutide-treated groups (5, 10, and 20 μg) on days 7 and 10. Even on the 10th day, the therapeutic effect of 20 μg liraglutide was superior to that of dexamethasone (20 μg). Liraglutide treatment successfully improved the severity score of synovitis in an MIA mouse model and significantly reduced the secretion of IL-6, PGE2, and nitric oxide in chondrocytes and macrophages. Correlation analysis indicated that there was a 91% correlation between the synovitis score and the results of the von Frey test. Therefore, the authors believed that the analgesic effect may be mediated at least partially by the anti-inflammatory effect of liraglutide. Clinical trials further supported the results of animal experiments. A cohort analysis of patients with OA from China revealed the benefits of GLP-1RA in OA. Postmedication analysis of 1807 patients with knee OA combined with type 2 diabetes (including 233 GLP-1RA-used patients and 1574 non-GLP-1RA-used patients) revealed that pain was relieved in the GLP-1RA group, as analyzed by the Western Ontario and McMaster Universities Osteoarthritis Index (WOMAC) total and pain subscale scores. The rate of cartilage loss in the medial femorotibial joint was significantly reduced, the consumption of symptom-relieving medication decreased, and the incidence of knee joint surgeries decreased.^[22]^ Recently, a randomized, double-blind, placebo-controlled trial involving 407 patients with both obesity and knee OA conducted at 61 sites in 11 countries demonstrated that once-weekly subcutaneous 2.4 mg semaglutide reduced body weight, alleviated knee OA-related pain, and decreased the use of nonsteroidal anti-inflammatory drugs. The results showed that at week 68, compared with the baseline, body weight decreased significantly (−13.7% with semaglutide vs −3.2% with placebo, *P* < .001), while the WOMAC pain score decreased significantly (−41.7 with semaglutide vs −27.5 with placebo, *P* < .001).^[23]^ Another clinical study reported that dulaglutide treatment enabled better blood glucose management, less knee pain, and weight loss in 98 older patients with OA combined with type 2 diabetes.^[24]^

However, studies have also reported that the use of GLP-1RA has not improved the pain in animals and humans. In the LPS-induced acute synovitis equine model, researchers used a special pain quantification method to conduct gait analysis on the asymmetric response of equine head movement in trots and then quantified lameness. The results showed that 6 hours postunilateral intra-articular liraglutide treatment, compared to the untreated joint, there was no significant difference in equine head movement asymmetry and negligible effects on inflammatory markers or in markers for cartilage metabolism.^[13]^ The results of another randomized controlled trial showed that after adding an 8-week prerandom assignment diet, compared with the placebo group, continued administration of liraglutide for 52 weeks induced significant weight loss, but did not alleviate knee pain in patients who were overweight/obese with knee OA.^[21]^

Researchers hypothesized that the observed clinical benefits might mainly result from reduced joint load mediated by weight loss,^[22,23]^ or the body’s metabolism affected by lower blood sugar,^[24]^ rather than the direct analgesic effect of the drug.^[29]^ The ambiguity regarding GLP-1RA-induced analgesia observed in different studies may be attributed to the differences in drug dosage, administration method, species specificity, potential comorbidities, and follow-up time. Therefore, further clinical and mechanism studies are required to confirm these findings.^[25]^

## 3. Application of GLP-1RA in OP

During bone metabolism, the imbalance between bone formation and bone resorption leads to the occurrence and development of OP, thereby increasing the risk of fractures. Osteoblasts and osteoclasts are 2 important cells that maintain bone homeostasis. GLP-1R is expressed in osteoblasts^[30]^ and osteoclasts,^[31]^ which indicates that GLP-1 may play a role in bone remodeling. Several studies have found that GLP-1RA has the potential to improve OP for various reasons, including diabetic OP,^[32]^ elderly OP,^[33]^ postmenopausal OP,^[34]^ and glucocorticoid-induced OP.^[35]^ The following text will discuss the influence of GLP-1RA on OP from 3 perspectives: promoting bone formation, inhibiting bone resorption, and reducing the incidence of fractures. Table 2 provide the specific contents and mechanisms.

**Table 2:** Effects of GLP-1RA on OP.

Drug	Model	Results	Conclusion	Reference
The impact on osteoblasts				
Liraglutide	Zucker diabetes rats	Ocn, Alpl, OPG, Runx2, P1NP, fracture load, BV/TV, stiffness↑; Tb.Sp, ROS, RAGE↓	Promote bone formation Increase bone strength	[32]
Liraglutide	Rat with glucocorticoid-induced osteoporosis	BMD, bone microstructure, bone biomechanical markers, ALP, OC, Tb.N, Tb. Th↑; Tb.Sp↓	Promote bone formation	[35]
Liraglutide	MC3T3-E1 preosteoblast	Col-1, ALP, OPG, Runx2, cAMP, AKT↑;	Promote bone formation	[36]
Liraglutide	Ovariectomized rats without diabetes	BV/TV, Tb.Th, Tb.N, BMD, bone mass, trabecular micro-architecture↑	Promote bone formation	[37]
Liraglutide	Bone marrow stromal cells (BMSCs)	Runx2, ALP, ColⅠ↑; RPRARγ↓	Promote bone formation	[37]
Liraglutide	Diabetic rats	Lipid deposition in bone marrow↓	Inhibit bone marrow adipogenesis	[38]
Liraglutide	ApoE−/−mice	PTH, AGEs, TRACP↓; BMD, BV/TV, Tb.Th:No change;	Improve bone metabolism	[39]
Liraglutide	Ovariectomized rats with streptozotocin-induced diabetes	BMD, OC, the thickness of trabeculae, total bone mass, OPG↑; RANKL↓	Promote bone formation	[40]
Liraglutide	Diabetes Goto-Kakizaki rat	BMD, BV/TV, Tb. Th, cortical BMD, cortical mean Thickness, cortical bone area/trabecular bone area, Runx2, ALP, COLL, OC, O PG↑; Tb.Sp↓	Promote bone formation	[41]
Semglutide	Rat bone marrow mesenchymal stem cells	cell proliferation and increase the proportion of G1 and S phase cells, OCN, RUNX2↑;	Affect osteogenic differentiation; Promote the proliferation of BMSCs.	[42]
Semglutide	Ovariectomized rat	BALP, trabecular bone, trabecular width, GSK3 β↓; BMD, calcitonin, PTH, vitamin D, trabecular area and width, cAMP, PKA, p-AKT, p-β-Catenin, BMP-2↑	Promote bone formation	[43]
Exenatide	Rat primary osteoblast	ALP, OPG, IGF-1, BPG↑; RANKL, p16, p23, p53↓	Promote the proliferation of osteoblasts	[33]
Exenatide	Ovariectomized mice	The number of osteoblasts, TGF-β1↑ The number of osteoclasts↓	Promote bone formation	[44]
Exenatide	Type 2 diabetic OLETF rats	BMD↑ Sclerostin↓	Inhibit sclerosing protein	[45]
Exenatide	Hindlimb-Unloading-Induced Bone Loss Rats	Bone mass, bone strength, BMD, BV/TV, Tb.N, Tb.Th, the number of osteoblast, Runx2, Balp, PPARγ, MAR, MS/BS, BFR/BS,↑		[46]
Exenatide	High-fat and high-calorie diet induced obesity in rats	BMD, BMC, OPG/RANKL ratio, BV/TV, Tb.Th.↑; Tb.Sp, ES/BS, BFR/BS↓	Promote bone formation	[47]
Exenatide	Streptozotocin induces type 2 diabetes in rats and insulin resistance in rats	OC, OPG, OPG/RANKL ratio, LRP5/DKK1 ratio, LRP5/SOST ratio↑; Tb.Sp,Tb.Pf, SMI↓	Promote bone formation	[48]
Exenatide	Aged ovariectomized rats	BMD, Tb.N, Tb. Th, bone strength, ALP, MAR, BFR, the number of osteoblasts, RUNX2, ALP, Col-1, OC, OPG↑;	Promote bone formation	[49]
Exenatide	Streptozotocin-induced type 2 diabetes Fructose-induced insulin-resistant rat	OC, OPG↑; Tb.Sp↓	Promote bone formation	[50]
The impact on bone resorption				
Liraglutide	Zucker diabetes rats	CTX-1↓	Inhibit bone resorption	[32]
Liraglutide	Rat with glucocorticoid-induced osteoporosis	TRACP, CTX-I↓	Inhibit bone resorption	[35]
Liraglutide	Mouse bone marrow-derived macrophages and RAW264.7	The number of TRAP-positive multinucleated osteoclasts, the osteoclast absorption area TRACP, Cathepsin K, NFATc1↓	Inhibit bone resorption	[51]
Liraglutide	Ovariectomized rats with streptozotocin-induced diabetes	CTX-1, osteoclast number↓	Inhibit bone resorption	[40]
Semglutide	Ovariectomized rat	Osteocalcin,RANKL, Ca2+, RANKL, CTX-I, PYD↓ IL-6↓	Inhibit bone resorption	[43]
Exenatide	Aged ovariectomized rats	CTX-1, the number of osteoclasts, RANKL, Tb.Sp, Conn.D, SMI, DPD/creatinine ratio↓	Inhibit bone resorption	[49]
Exenatide	Streptozotocin-induced type 2 diabetes Fructose-induced insulin-resistant rat	The OPG/RANKL mRNA ratio increased from 0.9 to 1.5, and from 0.81 to 1.25	Inhibit bone resorption	[50]
The impact on fractures				
Lxisenatide Liraglutide		Fracture risk↓	Treatment takes more than 52 weeks to significantly reduce fracture risk	[52]
Liraglutide	Ovariectomy induced osteoporosis in female Sprague-Dawley rats	Fracture line, the number of osteoclasts, the number of osteoclasts↓ BMD, callus area, thickness, and sagittal cross-section of femur↑;	Promote fracture healing	[53]
Liraglutide	--	--	Reduce risk of fractures	[54]
Liraglutide	Patients with diabetes and nondiabetes	Fracture incidence rate↓(0.21% vs 0.59%)	Reduce risk of fractures	[55]
		Fracture incidence rate in the exenatide group vs control group (0.74% vs 0.26%)		
Exenatide	Type 2 diabetes patients	Lowest risk of fracture	Reduce risk of fractures	[56]
Exenatide	--	--	Increase risk of fractures	[54]

AGE = advanced glycation end products, AKT = protein kinase B, ALP = alkaline phosphatase, ALPL = alkaline phosphatase, liver/bone/kidney isozyme, BALP = bone alkaline phosphatase, BFR/BS = bone formation rate, BMD = bone mineral density, BMP-2 = bone morphogenetic protein-2, BPG = 2,3-bisphosphoglycerate, BV/TV = bonevolume fraction, cAMP = cyclic adenosine monophosphate, Col-1 = collagen 1, CTX = C-terminal telopeptides of typeⅠcollagen, DPD = deoxypyridinoline, ES/BS = eroded surface per bone surface, GSK3β = glycogen synthase kinase 3 Beta, IGF-1 = insulin-like growth factor-1, MAR = mineral apposition rate, NFATc1 = nuclear factor of activated T cells, OC/Ocn = osteocalcin, OPG = osteoprotegerin, P1NP = procollagen I N-terminal propeptide, p-AKT = phosphorylated AKT, PKA = protein kinase A, PPAR-γ = peroxisome proliferator-activated receptor gamma, PTH = parathyroid hormone, PYD = pyridinoline, RAGE = receptor for advanced glycation end products, RANKL = receptor activator for nuclear factor-κ b ligand, ROS = reactive oxygen species, Runx2 = runt-related transcription factor 2, SMI = structure model index, Tb.N = trabecular number, Tb.Pf = trabecular bone pattern factor, Tb.Sp = trabecular separation of bone, Tb.Th = trabecular thickness, TGF-β = transforming growth factor-β, TRACP = tartrate-resistant acid phosphatase.

### 3.1. GLP-1RA promotes bone formation

Osteoblasts, which originate from mesenchymal stem cells and are the main functional cells involved in bone formation and are responsible for the synthesis, secretion, and mineralization of bone matrix. Studies have shown that GLP-1RA can promote osteoblast differentiation and bone formation through multiple signaling pathways after GLP-1R activation. In a macrophage-depleted model, exendin-4 (exenatide) promoted the polarization of bone-marrow-derived macrophages into the M2 subtype, increased the secretion of transforming growth factor-β, and induced the migration of bone mesenchymal stem cells (BMSCs) to the bone surface via the PKA-STAT3 signaling.^[44]^ Liraglutide acted directly on mouse MC3T3-E1 preosteoblasts through GLP-1R, activated the phosphatidylinositol 3-kinase/protein kinase B, extracellular regulated protein kinase, or cAMP/PKA signaling pathways, and increased the nuclear accumulation of β-catenin. The expression of osteoblast biomarkers, such as alkaline phosphatase, type I collagen (Col I), runt-related transcription factor 2, and osteocalcin, was upregulated at different stages. This promotes the osteoblasts proliferation and matrix mineralization.^[36]^ Semaglutide upregulated the expression of BMD, alkaline phosphatase, and runt-related transcription factor 2, promoted the proliferation and osteogenic differentiation of rat BMSCs by activating the wingless-type/integrated/low-density lipoprotein receptor-related protein 5/beta-catenin signaling pathway, and simultaneously reduced the incidence of bone turnover.^[42]^ The key role of β-catenin in the bone protective effect of semaglutide was demonstrated in an ovariectomized rat model of OP.^[43]^ Sclerostin was an inhibitor of the Wnt/β-catenin pathway and inhibited osteoblast activation by binding to the LRP5/6 receptor. Exenatide inhibited the mRNA and protein expression of SOST/sclerostin in osteocyte-like MLO-Y4 cells, alleviated the inhibitory effect on osteoblasts, and promoted bone formation.^[45]^

GLP-1RA can further promote osteogenic differentiation by blocking adipose differentiation.^[37,38,46,47]^ Liraglutide (10 nM) reduced the expression of peroxisome proliferator-activated receptor-γ and inhibited fat synthesis.^[37]^ Some researchers have found that liraglutide may reduce bone marrow fat accumulation by inactivating the miR-150-5p/GDF11 axis in diabetes.^[38]^ Exendin-4 may activate the GLP-1 receptor to inhibit adipogenic differentiation of BMSC.^[46]^ After subcutaneous administration of exendin-4 (0.1 nmol/kg/h) for 3 consecutive days, it reversed osteopenia in rats on a hyperlipidemic diet (35 days).^[47]^

### 3.2. GLP-1RA inhibits bone resorption

Osteoclasts are large multinucleated cells in the skeletal system that are responsible for breaking down bone tissue and originate from the blood mononuclear macrophage system. They can dissolve minerals and collagen in bones by secreting acidic substances and enzymes, and are the main functional cells of bone resorption. Multiple studies have confirmed that GLP-1RA promotes bone formation and inhibits osteoclast function and bone resorption. Liraglutide significantly reduced AGEs in ApoE^−/−^ mice and inhibits bone resorption.^[39]^ Through the analysis of 17 studies, liraglutide could partially improve changes in imaging and bone pathology, increase serum osteocalcin (OC) and *N*-terminal propeptide of type I procollagen, and reduce the C-terminal cross-linked terminal peptide of serum type I collagen, regardless of the presence of diabetes. The main mechanism of action is to promote the activation of osteoblast proliferation via the Wnt signaling pathway and inhibit the activation of osteoclasts *via* the osteoprotegerin (OPG)/receptor activator for nuclear factor-κ b ligand (RANKL)/RANK signaling pathway.^[57]^ Li et al^[51]^ found that liraglutide treatment of BMMs and RAW264.7 cells could inhibit the formation of osteoclasts and bone resorption. The specific mechanism is that liraglutide inhibited the activation of NF-κβ and mitogen-activated protein kinases signaling pathways, reduced the level of nuclear factor of activated T cells (NFATc1), and inhibited the nuclear penetration of NFATc1, thereby suppressing the expression of osteoclast-related genes mediated by NFATc1, such as tartrate-resistant acid phosphatase and cathepsin K.

Most studies have suggested that exenatide, liraglutide, and semaglutide increase the OPG/RANKL ratio, thus reducing osteoclast production.^[40,47–49]^ However, studies insist on the opposite opinion. Studies have found that liraglutide can simultaneously upregulate the expression of OPG and RANKL; therefore, no significant change exists in the OPG/RANKL ratio.^[41]^ Exenatide did not significantly alter the serum level of tartrate-resistant acid phosphatase 5b (a marker of osteoclast activity). Researchers believe that exenatide mainly increases bone density by promoting bone formation rather than inhibiting bone resorption.^[45,50]^

Calcitonin effectively inhibits bone resorption. GLP-1RA can increase plasma calcitonin levels and inhibit bone resorption.^[43,58–60]^ However, Pereira et al^[34]^ found that although exenatide increased serum calcitonin, liraglutide did not affect plasma calcitonin. The reason for the contradiction in the experimental results might be the difference in species. Rodents have a greater number of C cells (thyroid tissue), and GLP-1RAs can activate the receptors on these cells and stimulate the release of calcitonin. In contrast, primates, including humans, have significantly fewer C cells, and the physiological effect of calcitonin is relatively limited. Clinical studies have found that in patients with type 2 diabetes, even after 2 years of liraglutide treatment, calcitonin levels remain within the lower limit of the normal range. Therefore, during the research process of GLP-1RA, special attention should be paid to species specificity. However, the results of rodent research cannot be directly applied to humans.^[60]^

### 3.3. GLP-1RA reduces the incidence of fracture

Fractures are the greatest risk of OP. The disability and mortality rate of patients with fractures have increased significantly. The risk of OP is evaluated based on BMD. Scholars hold different opinions on whether GLP-1RA can reduce the risk of fractures. Liraglutide and lixisenatide were associated with a reduced risk of fractures, and these beneficial effects depended on the treatment duration.^[52]^ A meta-analysis of randomized controlled trials further confirmed that liraglutide caused a time-dependent reduction in the incidence of fractures in patients with type 2 diabetes, and the observed protective effect became more significant with increasing treatment time.^[8]^ Liraglutide improved bone healing in rats with oophorectomy-induced osteoporotic fractures. Callus formation was enhanced, trabecular arrangement was more regular, collagen fibers were more abundant, the number of osteoclasts was reduced, and femoral stiffness and ultimate load were significantly increased.^[53]^ Zhang et al^[56]^ conducted a meta-analysis of clinical trials and found that Exenatide can prevent fractures in patients with diabetes.

In contrast to previous reports, Su et al^[54]^ found that exenatide increased the risk of fractures. Furthermore, some researchers have found that liraglutide and exenatide do not cause fractures.^[52,61–64]^ Further research found that BMD stability in the liraglutide group was not related to weight changes, while the increased risk of fractures related to exenatide might be partly attributed to bone loss induced by significant weight loss. The exenatide group experienced more frequent hypoglycemic events, which may have increased the risk of falls. However, the meta-analyses lacked sufficient data on fall events. Therefore, future studies using fractures as the primary endpoint will require longer follow-up periods.^[54]^

Dipeptidyl peptidase 4 inhibitors (DPP-4i) have been reported to reduce the risk of fractures.^[65,66]^ DPP-4i prolonged the effect of GLP-1 and promoted bone anabolism.^[67]^ Experimental studies have shown that GLP-1RA is more effective than DPP-4i in bone protection.^[55]^ However, researchers had also suggested that GLP-1RA is inferior to DPP-4i, and the lumbar BMD of patients who switched from DPP-4i to GLP-1RA decreased.^[68]^ Regarding the inconsistency in the results, we speculate that this might be due to different drugs, different doses, durations, and individual differences. Further research is required to elucidate the effects of different GLP-1RAs on bone formation.

## 4. Summary and limitations

GLP-1 is an important hormone secreted by intestinal cells. This study systematically reviews the important roles of GLP-1 agonists in the treatment of OA and OP. GLP-1RA was discovered to be promising for the treatment of OA, reversing the development of OA through its anti-inflammatory action, affecting chondrocyte matrix metabolism, and inhibiting endoplasmic reticulum stress, oxidative stress, and analgesia. Second, GLP-1RA alleviates OP symptoms by promoting osteoblast differentiation and inhibiting osteoclast resorption. Moreover, the greatest risk of OP is fracture, and there are different views on whether GLP-1RA can reduce the risk of fracture, and even the same drug appears to lead to different conclusions. The conflicting results are hypothesized to be related to the treatment time, dose, and individual differences, which should be investigated further.

Numerous gaps exist in research on GLP-1 in OA and OP, and research in OA and OP is still in its infancy. The majority of the currently available findings are from artificial rodent models, and few effects of GLP-1RA on the 2 diseases have been found in humans and alternative large animal species. Clinical studies have identified persistent uncertainties regarding the direct articular effects, long-term efficacy, and safety profiles of these interventions in heterogeneous populations of patients with OA and OP. In the future, large-scale clinical trials are required to assess the effects of GLP-1RA on bone metabolism and to gain a deeper understanding of the efficacy and safety of this class of medicine in patients with OA and OP.

## Author contributions

**Writing – original draft:** Meiqi Zheng.

**Investigation:** Meiqi Zheng, Jingjing Zhao, Yuxuan Wang.

**Validation:** Zifan Cui, Zihong Qiao, Hongzhuo Wu.

**Methodology:** Meiqi Zheng, Chenxia Shi, Xiaofeng Wang.

**Visualization:** Meiqi Zheng, Jingjing Zhao.

**Supervision:** Chenxia Shi, Xiaofeng Wang.

**Writing – review & editing:** Chenxia Shi, Xiaofeng Wang.
